# Single-cell differential splicing analysis reveals high heterogeneity of liver tumor-infiltrating T cells

**DOI:** 10.1038/s41598-021-84693-w

**Published:** 2021-03-05

**Authors:** Shang Liu, Biaofeng Zhou, Liang Wu, Yan Sun, Jie Chen, Shiping Liu

**Affiliations:** 1 BGI Education Center, University of Chinese Academy of Sciences (UCAS), Shenzhen, 518083 China; 2 BGI-Shenzhen, Beishan Industrial Zone, Shenzhen, 518083 China; 3 Shenzhen Key Laboratory of Single-Cell Omics, China National GeneBank, Shenzhen, 518120 China

**Keywords:** Software, Tumour heterogeneity, Transcriptomics, RNA splicing

## Abstract

Recent advances in single-cell RNA sequencing (scRNA-seq) have improved our understanding of the association between tumor-infiltrating lymphocyte (TILs) heterogeneity and cancer initiation and progression. However, studies investigating alternative splicing (AS) as an important regulatory factor of heterogeneity remain limited. Here, we developed a new computational tool, DESJ-detection, which accurately detects differentially expressed splicing junctions (DESJs) between cell groups at the single-cell level. We analyzed 5063 T cells of hepatocellular carcinoma (HCC) and identified 1176 DESJs across 11 T cell subtypes. Interestingly, DESJs were enriched in UTRs, and have putative effects on heterogeneity. Cell subtypes with a similar function closely clustered together at the AS level. Meanwhile, we identified a novel cell state, pre-activation with the isoform markers ARHGAP15-205. In summary, we present a comprehensive investigation of alternative splicing differences, which provided novel insights into T cell heterogeneity and can be applied to other full-length scRNA-seq datasets.

## Introduction

T cell heterogeneity in the tumor microenvironment (TME) is tightly linked to tumor progression, prognosis, and therapeutic efficacy. Systematic interrogation of tumor-infiltrating lymphocytes has been performed in liver^1^, lung^2^, colon^3^ and breast^4^ cancers using scRNA-seq. Effector and cytotoxic T cells can exert anti-tumor effects by targeting tumor cells, and levels of effector CD8^+^ T cells are predictive of good survival in several cancers^5–7^. However, tumor-infiltrating regulatory T cells (Tregs) suppress the activity of T cells, myeloid cells, and stromal cells^8^ through different mediators including FOXP3. Immunosuppressive cytokines activate co-inhibitory receptors on T cells such as PD1 and CTLA4, thus driving T cell dysfunction and exhaustion^9^. Meanwhile, the function of these immunosuppressive cytokines and co-inhibitory receptors is influenced by alternative splicing. For example, one of the isoforms of *FOXP3* lacking exon 2 and exon 7 cannot perform its immunosuppressive function^10^ and a soluble *CTLA4* isoform exhibits different effects on the T cell state compared to the full-length *CTLA4* isoform^11^. Therefore, investigating the influence of AS on the T cell state in TME will further our understanding of T cell heterogeneity and the development of cancer therapies.

Alternative splicing analysis based on scRNA-seq is revolutionizing our understanding of the effect of AS on immune cells. Recently, scRNA-seq revealed the bimodality of AS in immune cells, and bulk RNA-seq might mask differences in AS between single cells^12^. However, the current computational framework for RNA-seq AS analysis does not effectively detect differential splicing between groups at the single-cell level. DEXSeq^13^, rMATS^14^, and MISO^15^ were developed for bulk RNA-seq data. Therefore, these methods might lead to incorrect results as the underlying algorithms may not be appropriate to process scRNA-seq data due to the low sequencing depth and high dropout rate. Some programs, BRIE^16^, VALERIE^17^, Millefy^18^, Outrigger^19^, and an NMF-based method^20^, were recently developed to process scRNA-seq data. However, BRIE requires performing a pairwise comparison between every two cells to detect differential splicing, which is time-consuming and impractical. Outrigger utilizes the distribution mode of percent-spliced-in (Psi) to detect differential splicing between cell groups. However, the distribution modes are limited to five types, and do not accurately reflect reality. Thus, there is an urgent need to develop a convenient and effective computation tool to detect differential splicing between groups.

To explore T cell splicing heterogeneity in high resolution, we developed a novel computation framework, DESJ-detection, to detect differential splicing between groups at the single-cell level. We applied it to a published scRNA-seq dataset from HCC patients. We identified 1176 DESJs across the 11 cell clusters and found that functionally similar T cell subsets shared a similar splicing pattern. DESJs were enriched in UTRs, and play a potential role in heterogeneity. We revealed a relationship between AS and T cell functional subpopulations, with a focus on pre-activation subpopulations. We also validated our findings in a single cell dataset from CRC patients. Thus, systematic evaluation of differential splicing across T cells in TME of HCC furthers our understanding of the AS characteristics of TILs and will facilitate improvements to cancer diagnosis and treatment.

## Results

### An overview of DESJ-detection

**Figure 1:**
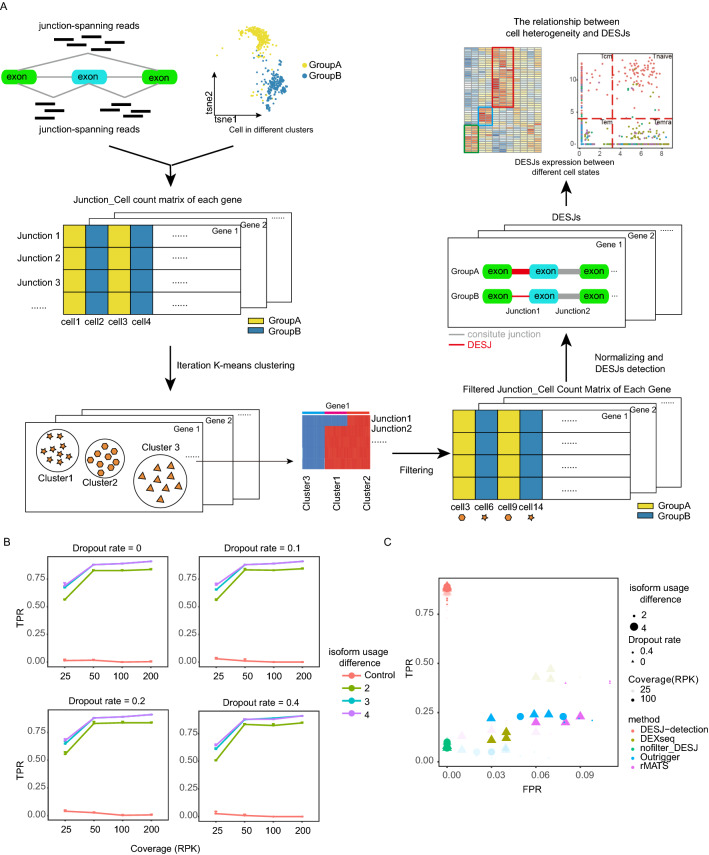
An overview of DESJ-detection. (**A**) DESJ-detection applies iterative k-means and gene-level normalization to filter cells and weaken the interference of gene expression. It provides a specific DESJ for each cell group. (**B**) A point plot demonstrating the TPR (true positive ratio) of DESJ-detection under different coverages, dropout rates, and isoform usage differences. (**C**) TPR, FPR (false positive ratio) and TP (true positive) are estimated by DESJ-detection and four other methods under different coverages, dropout rates, and isoform usage differences. Isoform usage difference refers to differences in the fraction of total expression of a gene represented by the expression of each of its isoforms.

Revealing splicing differences at the single-cell level would deepen our understanding of cell heterogeneity, function, and phenotype. Some major challenges of differential splicing analysis at the single-cell level include that scRNA-seq data has a high rate of dropout events and low sequencing depth compared to bulk RNA-Seq. These two features hinder our ability to accurately reveal the splicing structure of genes. In addition, splicing analysis is mainly limited to exon skipping (SE) and mutually exclusive exons (MXE). To address these challenges, we proposed DESJ-detection, an algorithm that uses junction-spanning reads to detect DESJs (Fig. 1A). First, we input all the junction read counts of each cell and output a junction-cell count matrix for each gene. Second, we applied iterative K-means to cluster cells and removed the clusters with low expression (standard deviation < 0.2 and mean < 1) of all junctions resulting from low coverage and high dropout rate. Next, we utilized a new normalization method at the gene level to eliminate the interference of DEGs on DESJ detection. Specifically, this normalized the junction read count with the read count of each gene rather than uniquely mapped reads of each cell. Finally, we identified DESJs based on the Limma-tread algorithm with fold change and adjusted *p*-values. DESJ-detection can detect DESJs at any regions of a given gene; therefore, it can discover any type of AS, rather than being limited to SE and MXE events. We also developed a convenient pipeline (https://github.com/liushang17/DESJ-detection), which covers the generation of junctions, filtering and annotation of junctions, preparation of junction count matrices, and detection of DESJs (Fig. S1A).

To assess the performance of the software in terms of DESJ detection, we simulated scRNA-seq data with a pipeline based on Spanki considering different factors including read coverage, dropout rate, and isoform usage difference. Our method proved to be effective. For example, the simulated cells were divided into five clusters by the expression of two isoforms of PPT1. Four cell clusters showed differential junction expression and another cluster with low gene expression was removed by iterative K-means clustering (Fig. S1B). We observed sensitivity up to ~ 70%, even at the lowest coverage level (RPK = 25) when the isoform usage difference is more than the control and without dropout events (Fig. 1B). The sensitivity was essentially maintained at 85% at the general coverage level (RPK >  = 50). In addition, the sensitivity exceeds 70% when the dropout rate is < 0. Over 95% of identified genes exhibited DESJ. Further, we also evaluated DESJ-detection by comparing it with other software including Outrigger, DEXSeq, rMATs, and Limma-trend (Fig. 1C). DESJ-detection performed the best (high TPR and low FPR) under all conditions tested. Outrigger had similarly low FPR but divergent TPR, and was especially influenced by coverage. High coverage led to better performance in Outrigger. rMATS and DEXseq were heavily influenced by dropout events. When the dropout ratio = 0.4, no gene was detected in rMATs, and DEXseq failed to run successfully. Limma-trend exhibited lower TPR but similar FPR to that of DESJ-detection. We also applied DESJ-detection to a dataset, which contain 63 iPSCs, 73 NPCs, and 70 MNs^19^. The differential usage of exon 9 and exon 10 of PKM gene, could be detected by DESJ-detection (Fig. S1C). Taken together, DESJ-detection is robust and highly sensitive to DESJs.

### Differential usage of junctions in UTRs across T cell clusters

We performed DESJ-detection on a published scRNA-seq data set^1^. This dataset included 5063 T cells from tumor tissues, normal tissues, and peripheral blood of six HCC patients that had been assigned to 11 T cell subsets including naïve T cells (C01_CD8.LEF1, C06_CD4.CCR7), effector T cells (C02_CD8.CX3CR1, C11_CD4.GNLY), exhausted T cells (C04_CD8.LAYN, C10_CD4.CXCL13), Tregs (C07_CD4.FOXP3, C08_CD4.CTLA4), mucosal-associated invariant T cells (C03_CD8.SLC4A10), and intermediate T cells (C05_CD8.GZMK, C09_CD4.GZMA). We obtained a set of 134,414 junctions that were characterized by read counts < 4 in at least 10 cells, covering 12,587 genes (Fig. S2A and Fig. S2B). The junctions that were annotated to one gene were retained. In the end, we retained 119,311 junctions from 10,556 genes. Using DESJs analysis, we identified 1176 DESJs across 11 clusters (log2(FC) ≥ 1, adjusted *p*-value ≤ 0.01; Supplementary Table2).

**Figure 2:**
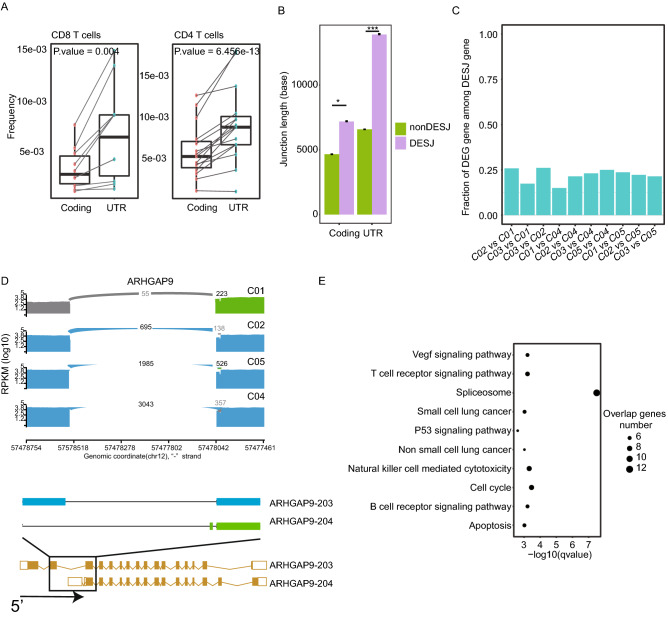
Differential usage of junctions in the UTR regions across T cell clusters. (**A**) Frequency of DESJ in the UTR regions is significantly higher than in the coding regions. These points represent T cell clusters. (**B**) Length difference between DESJ and non-DESJ in the coding region and the UTR region. (**C**) A bar plot depicting the fraction of DEG genes among DAS genes across CD8^+^ T cells. (**D**) Sashimi plots illustrating the read distribution of ARHGAP9 in CD8^+^ T cells from P0508. The colors represent different isoforms. This alternative splicing of ARHGAP9 happens in the 5′ UTR regions. Naïve T cells (C01_CD8.LEF1) show obvious differential usage of isoforms compared to other clusters. (**E**) Results of KEGG pathway analysis of genes with differential splicing in the UTR regions.

To characterize the distribution of DESJs across the genome, we investigated the frequency of DESJs in different genomic regions. We found a significant higher frequency of DESJs in UTRs than in coding regions between clusters (*p*-value = 0.004 for CD8^+^ T cells and 6.456e−13 for CD4^+^ T cells; Student’s *t* test; Fig. 2A). AS in the 5′ UTR occurs more frequently than in the 3′ UTR (Fig. S2C), in line with the findings from previous studies^21^. There are similar phenomena in the human reference transcriptome. A junction is considered to be involved in alternative splicing when this junction does appear in some isoforms of the gene, but not in all isoforms of the gene. AS in the UTRs (98.7%) occurs potentially more frequently than in the coding regions (83.6%). Total 6115 AS junction happened in 5′ UTR while 4946 in 3′ UTR. AS events in UTRs might involves TTSs and TSSs (Fig. S2E). Higher frequency of DESJs in UTRs may be due to longer junction lengths in UTRs. Junction length refers to the genomic position of the last base of the intron minus the first base (Fig. S2F). We additionally observed that DESJs are significantly longer than non-DESJ in both UTRs and coding regions. The DESJs in UTRs were also longer than those in coding regions (Fig. 2B). UTRs are usually longer than coding regions. Thus, these two phenomena might be explained by the fact that longer junctions would provide more possible splice sites and potential regulatory regions. Therefore, our results highlight the generality of AS in UTRs.

In the meantime, we noticed that a few DEGs between clusters were also DESJ genes in T cells (Fig. 2C). The proportion ranged from 11–37% across T cell clusters, which is similar to findings from a previous study^22^. For example, *ARHGAP9*, a member of RhoGAP family that is associated with good prognosis, was a differential expressed gene (highly expressed in C04_CD8.LAY, C10_CD4.CXCL13, and C08_CD4. CTLA4), and also showed differential splicing in UTRs across CD8^+^ T cell clusters (C01_CD8.LEF1, C02_CD8.CX3CR1, C04_CD8.LAYN, C05_CD8.GZMK). Specifically, ARHGAP9-203 was upregulated in exhausted T cells and tumor-infiltrated Tregs, while ARHGAP9-204 was mainly expressed in naïve T cells and peripheral blood Tregs (Fig. 2D). Therefore, our results indicate that AS in UTRs may play a role in regulating gene expression between cell clusters.

The Kyoto Encyclopedia of Genes and Genomes (KEGG) pathway analysis of genes with DESJs in their UTRs revealed involvement of the VEGF signaling pathway, T cell receptor signaling pathway, spliceosome, and P53 signaling pathway^23–25^ (Fig. 2E). Meanwhile, the genes with DESJs in the coding region were associated with innate immune pathways and spliceosomes (Fig. S2G). This emphasizes that AS in UTRs may be related to the specific function(s) of cells. Taken together, AS in UTRs is common and may contribute to the regulation of gene expression and cell heterogeneity.

### T cell heterogeneity at the splicing level

**Figure 3:**
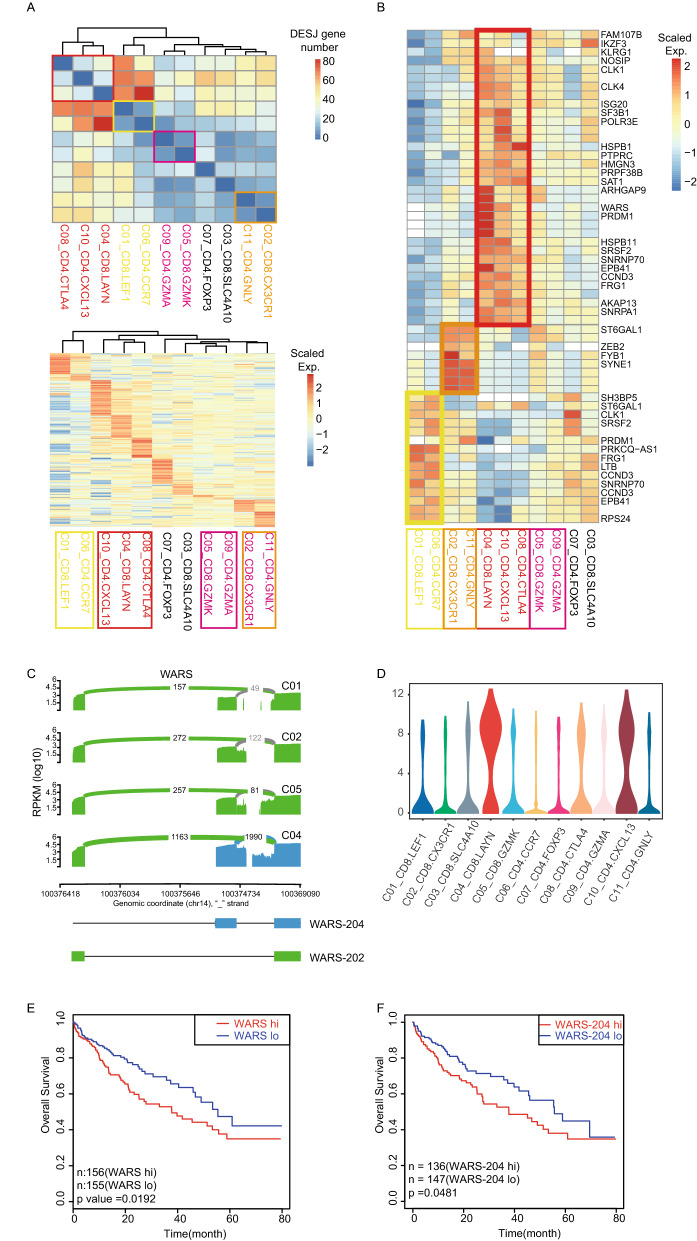
T cell heterogeneity at the splicing level. (**A**) Upper: Heatmap of DESJ gene number between pairwise clusters across T cells. Below: z-score normalized mean expression of all DESJ in each T cell cluster. Boxes with different colors highlight the patterns of different functional T subtypes. (**B**) z-score normalized mean expression of selected DESJ with similar functions. (**C**) Sashimi plots illustrating the read distribution of WARS in CD8^+^ T cells from the P0508 patient. WARS-204 is highly expressed in exhausted T clusters (C04_CD8.LAYN). (**D**) Violin plots comparing the expression of WARS among 11 T cell clusters. (**E**) A disease-free survival (DFS) curve based on TCGA HCC data showing that patients with higher expression of WARS had a poor prognosis. (**F**) DFS curve based on the TCGA HCC cohort showing that higher expression of WARS-204 in tumor is associated with bad prognosis.

To explore the association between AS and the function of T cell heterogeneity, we examined DESJs across T cell clusters to obtain cell-type-specific DESJs. In this study, we detected 335 DESJs from 165 genes among CD8^+^ sub-clusters and 484 junctions from 239 genes among CD4^+^ sub-clusters (Supplementary Table 2). We used two distinct indices to hierarchically cluster T cells, the number of DESJ genes and the expression of DESJs across all cell clusters. Both indices revealed that cells with a similar function rather than lineage exhibited a similar AS pattern. (Fig. 3A). For example, tumor-infiltrating Tregs (C08_CD4.CTLA4, C10_CD4.CXCL13) and exhausted T cells (C04_CD8.LAYN) clustered together, demonstrating a huge difference between these cells and others. In addition, naïve T cells (C01_CD8.LEF1, C06_CD4.CCR7), effector T cells (C02_CD8.CX3CR1, C11_CD4.GNLY), and intermediate state T cells (C05_CD8.GZMK, C09_CD4.GZMA) clustered together respectively. We also utilize a published scRNA-Seq dataset, which contains 11,138 T cells from tumor tissues, normal tissues, and peripheral blood of 12 CRC patients^3^, to further validate our findings. The hierarchically clustering of the number of DESJ genes and the expression of DESJs across all cell clusters, is similar to HCC, indicating that cells with a similar function exhibited a similar AS pattern. (Fig. S3A). Exhausted T cells in both CRC and HCC showed the highest number of DESJs compared to other T cells, indicating that exhausted T cells exhibit the greatest changes in AS. These results demonstrated that junction usage differences between cell clusters mainly depends on the functional state of the clusters.

We next focused on the DESJ genes in four functional states including naïve T cells, effector T cells, exhausted T cells, and intermediate T cells. Naïve and exhausted T cells mainly showed differential splicing in genes relating to splicing and immunity such as *CD45*, *HSPB1*, *CLK1*, *SRSF2*, *SNRNP70*, *PRDM1*, *NOSIP*; effector T cells were characterized by differential splicing in *ZEB2*, *FYB1*, and *SYNE1* (Fig. 3B). The DESJs and DESJ genes between CRC and HCC show a significant overlap (Fig. S3B). Meanwhile, DESJ genes between clusters with different states in HCC shares most with DESJ genes between the clusters with the similar states in CRC (Fig. S3C). These genes, which shows the differential splicing across T cells population in HCC, also appear in T cells in CRC, including *CD45, CLK1, SNRNP70, ZEB2, FYB1,* and *SYNE1* (Fig. S3B). Specially, *PRDM1* and *NOSIP* shows differential splicing between exhaustion T cells and non-exhaustion T cell, not only in CRC and HCC, also appearing in non-small-cell lung cancer^26^. *WARS* was highly expressed in exhausted T cells and is a marker of exhaustion that showed differential splicing between exhausted T cells and other T cells (Fig. 3C,D; Fig. S4A). The junction representing WARS-202 (chr14_100369259_100376259_2) showed widespread expression in all T cells while the junction representing WARS-204 (chr14_100369259_100375282_2) was widely expressed in Tregs (C08_CD4.CTLA4) and exhausted T cells (C04_CD8.LAYN, C10_CD4.CXCL13). These phenomes also appear in CRC datasets (Fig. S4E). Prognostic analysis using TCGA LIHC data revealed that elevated expression of WARS was associated with poor prognosis (Fig. 3E). We found that elevated expression of the WARS-204 isoform was correlated with poor prognosis (Fig. 3F). Prognostic analyses with the TCGA LIHC data at the isoform level also supported our results (Fig. S4B). We hypothesized that various immunity therapy-related target genes might also show this pattern, and identified several T cell immunity checkpoint genes whose elevated expression was related to poor prognosis, such as *TNFRSF4* (Fig. S4C). Expression of the two isoforms is mutually exclusive in T cells, rather than one isoform being more highly expressed than the other (Fig. S4D). In summary, these results demonstrate that AS significantly affects the function and phenotype of T cells and could be used as a potential marker for cancer prognosis and treatment.

### A novel functional subpopulation in activation state identified by ARHGAP15-205

**Figure 4:**
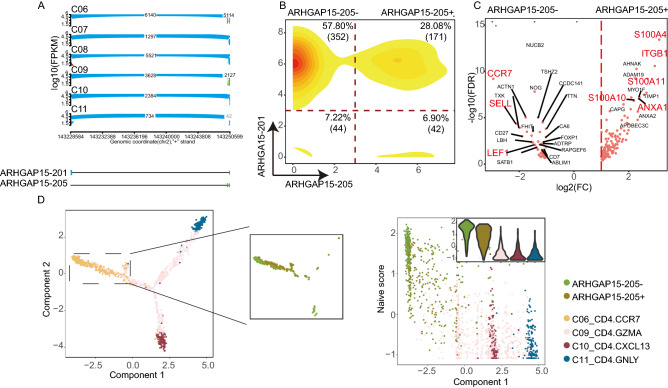
A novel functional subpopulation in activation state identified by ARHGAP15-205. (**A**) Sashimi plots illustrating the read distribution of ARHGAP15 in CD4^+^ T cells from patient P0508. ARHGAP15-205 is highly expressed in naïve T cells (C06_CD4.CCR7). (**B**) The bimodal distribution of ARHGAP15-205 shows the intrinsic heterogeneity in naïve T cells (C06_CD4.CCR7). Cell density is color-coded, with red denoting high density and yellow denoting low density. (**C**) Volcano plot showing DEGs between the ARHGAP15-205^+^ and ARHGAP15-205^−^ populations. Each red dot denotes an individual gene with an adjusted *p-*value < 0.01 (two-sided moderated *t*-test with limma) and fold change > |2|. (**D**) Left: CD4^+^ T helper cells were ordered along pseudotime in a two-dimensional state-space defined by Monocle2. Cell orders are inferred from the expression of DEGs across CD4^+^ T cell populations. Each point with different colors corresponds to individual cells in different clusters. The middle plot shows the order of the ARHGAP15-205^+^ and ARHGAP15-205^−^ populations. Right: The exhaustion score calculated by the mean expression of gene sets related to naïveness status correlated with Monocle components. Violin plots in the top corners show the distribution of naïveness scores in various cell clusters. Different colors represent different clusters. *p*-values were calculated by Pearson correlation, and *p* < 2.2 × 10^–16^ represents a *p-*value approaching 0.

To further reveal the heterogeneity of T cell clusters, we utilized DESJs to identify functional subpopulations. *ARHGAP15*, a Rac1-specific GAP, was reported to be associated with the development of diverse tumors, including colorectal cancer^27^, glioma^28^ and pancreatic ductal adenocarcinoma^29^. However, little is known about the relationship between T cell state and *ARHGAP15* at the isoform level. Our study discovered that ARHGAP15-201 was universally expressed in all cell clusters, but ARHGAP15-205 exhibited elevated expression in C06_CD4.CCR7 and C09_CD4.GZMA (Fig. 4A). Further, ARHGAP15-205 shows a striking bimodal expression distribution in both CD4 naïve T cells (C06_CD4.CCR7) and CD8 naïve T cells (C01_CD8.LEF1) (Fig. 4B; Fig. S5C). This implies that ARHGAP15-205 may affect the functional state of naïve T cells. We identified 174 genes that were highly expressed in ARHGAP15-205^+^ naïve T cells (FDR < 0.01, log2(FC) ≥ 1; Supplementary Table 1). These genes significantly overlapped with genes that are markers of an activated state as defined by previous studies (Fig. S5A). Thus, the ARHGAP15-205^+^ population may represent an activated state. Signature genes of ARHGAP15-205^+^ include *S100A4*, *ITGB1*, *S100A6,* and *LGALS1,* supporting that the ARHGAP15-205^+^ population trends towards an activated state (Fig. 4C). In contrast, the ARHGAP15-205^−^ population was characterized by high expression of genes related to a resting state including *CCR7*, *SELL,* and *LEF1*. Meanwhile, GO biological process enrichment analysis showed that the ARHGAP15-205^+^ population signature genes were enriched in cell differentiation (including leukocyte and lymphocyte differentiation) and cell activation (Fig. S5B). In addition, pseudotime analysis of cells in C06_CD4.CCR7, C09_CD4.GZMA, C10_CD4.CXCL13, and C11_CD4.GNLY showed that ARHGAP15-205^+^ cells clustered more closely to cells in C09_CD4.GZMA and had a lower naïve score compared with the ARHGAP15-205^−^ population (Fig. 4D). These results suggest that ARHGAP15-205^+^ CD4 naive T cells might be in the “pre-activation” state and possess immune killing function. Similar results were associated with respect to CD8 naïve T cells (C01_CD8-LEF1; Fig. S5C and Fig. S5D). In addition, ARHGAP15 also shows the differential splicing across CD4 T cells clusters in CRC, similar to HCC (Fig. S5E). ARHGAP15-205 shows a striking bimodal expression distribution in both CD4_C02.ANXA1 and CD8_C02.GPR183, which is similar to naïve T cell in transcriptome, but in central memory state (Fig. S5F). Furthermore, DEG analysis shows the ARHGAP15-205^+^ population in CD4_C02.ANXA1 clusters highly expresses *S100A4*, *ANXA1*, *S100A6,* and *LGALS1,* while ARHGAP15-205^−^ population was characterized by high expression of genes related to a resting state including *CCR7* (Fig. S5G). These results further supported our findings in HCC. In summary, ARHGAP15-205 may play a role in T cell activation.

We used Seurat to cluster cells C06_CD4.CCR7 with a TPM expression matrix. The clustering results based on gene expression were dissimilar to the classification results using AS of ARHGAP15 (Fig. S5H). Meanwhile, the expression distribution of ARHGAP15 also demonstrates that clustering based on gene may not identify the population in activation state (Fig. S5I). These indicate that the novel cell subtype may be indeed determined by AS. Altogether, these results emphasize that AS analysis at single-cell level would reveal cell heterogeneity and facilitate the discovery of cell sub-clusters in higher resolution than at the gene expression level.

## Discussion

scRNA-seq technology has developed rapidly and has been widely applied in many fields including tumor heterogeneity, cell differentiation, and neural development. Compared to 3′ enrichment methods, full-length single-cell RNA data can not only quantify gene expression but also analyze the structure of genes in high resolution, revealing features such as single nucleotide variants and AS events. Due to the lack of available software to analyze cell heterogeneity with AS, single-cell research is currently limited to gene expression profiling. Here we have developed software (DESJ-detection) for differential AS detection in full-length scRNA-seq datasets.

DESJ-detection was able to detect DESJs between different cell types at a single-cell level in a robust and effective manner. However, DESJ-detection could not accurately detect the isoform composition of a single cell for any given gene since some junctions may not uniquely belong to one isoform. Efforts to develop an improved version that addresses this shortcoming are ongoing, and will lead to the interpretation of isoform differences at a higher resolution.

We performed DESJs-detection in a T cell dataset from six patients diagnosed with HCC, which provided insight into T cell heterogeneity. Interestingly, cell clusters with a similar function displayed a low number of DESJ-related genes and possessed a similar DESJ expression pattern. These relationships may partly be because cells with a similar function would share similar expression profiles with respect to genes as well as isoforms. At the same time, some unique isoforms in exhausted T cells are related to poor prognosis, such as *WARS* and *CCND3*. Therefore, altering the isoform preference of specific genes in T cells may be an attractive avenue for improving cancer immunotherapy. Meanwhile, the association between AS and cell clusters may help infer the function of AS and predict novel subpopulations. For example, ARHGAP15-205 revealed a novel sub-cluster in T cell activation state. Further studies are needed to confirm these results by in vitro experiments, interrogate the underlying mechanisms, and identify other isoforms related to cell functional states.

With the rapid development of scRNA-seq, Smart-seq3 technology has emerged^30^, which is characterized by longer read length and faster sequencing. This would lead to a number of studies on single-cell AS, making it somewhat of a hot topic. However, the conditions to support single-cell AS analysis, including sequencing depth and coverage, have not been revealed. In addition, methods to construct AS profiles at the single-cell level are still lacking. Finally, combining single-cell AS and gene expression analyses has not been performed. We anticipate that our software will improve and enhance the study of AS.

## Methods

### Datasets

We downloaded the scRNA-seq raw reads of human T cells in fastq format from the EGD database (EGAS00001002072). The corresponding gene expression matrix was downloaded from the GEO database (GSE98638). This dataset contained 5063 T cells assigned into 12 clusters^1^. These T cells were sampled from peripheral blood, tumor, and adjacent normal liver tissue. Detailed clinical information about the patients and information on the cell clusters is listed in Table 1. The scRNA-seq raw reads of human T cells in fastq format was also downloaded from the EGD database (EGAS00001002791). The corresponding gene expression matrix was downloaded from the GEO database (GSE108989). This dataset contained 11,138 T cells from 12 patients with colorectal cancer, assigned into 20 clusters^3^. Detailed clinical information about the patients and information on the cell clusters is listed in Tables 1 and 2. The human genome (version GRCH38) was used as the reference genome for alignment with STAR (v2.5.3)^31^.

**Table 1:** Annotation about cell clusters in HCC datasets.

Cluster	Cell number	Function annotation	Type
C01_CD8.LEF1	161	Naïve T cell	CD8^+^ T cell
C02_CD8.CX3CR1	288	Effector T cell	CD8^+^ T cell
C03_CD8.SLC4A10	363	MAIT	CD8^+^ T cell
C04_CD8.LAYN	300	Exhausted T cell	CD8^+^ T cell
C05_CD8.GZMK	467	T cell in mediate state	CD8^+^ T cell
C06_CD4.CCR7	646	Naïve T cell	CD4^+^ T cell
C07_CD4.FOXP3	261	Peripheral Treg	CD4^+^ T cell
C08_CD4.CTLA4	582	Tumor Treg	CD4^+^ T cell
C09_CD4.GZMA	689	T cell in mediate state	CD4^+^ T cell
C10_CD4.CXCL13	146	Exhausted T cell	CD4^+^ T cell
C11_CD4.GNLY	167	Effector T cell	CD4^+^ T cell
Unknown	993	NA	NA

**Table 2:** Annotation about cell clusters in CRC datasets.

Cluster	Cell number	Function annotation	Type
CD8_C01.LEF1	164	Naïve T cell	CD8^+^ T cell
CD8_C02.GPR183	155	Central memory T cell	CD8^+^ T cell
CD8_C03.CX3CR1	773	Effector T cell	CD8^+^ T cell
CD8_C04.GZMK	363	Effector memory T cell	CD8^+^ T cell
CD8_C05.CD6	431	Resident memory T cell	CD8^+^ T cell
CD8_C06.CD160	363	Intraepithelial lymphocytes	CD8^+^ T cell
CD8_C07.LAYN	831	Exhausted T cell	CD8^+^ T cell
CD8_C08.SLC4A10	71	MAIT	CD8^+^ T cell
CD4_C01.CCR7	472	Naïve T cell	CD4^+^ T cell
CD4_C02.ANXA1	509	Central memory T cell	CD4^+^ T cell
CD4_C03.GNLY	170	Effector T cell	CD4^+^ T cell
CD4_C04.TCF7	331	Central memory T cell	CD4^+^ T cell
CD4_C05.CXCR6	639	Resident memory T cell	CD4^+^ T cell
CD4_C06.CXCR5	216	T follicular helper cell	CD4^+^ T cell
CD4_C07.GZMK	204	TH-1 like cell	CD4^+^ T cell
CD4_C08.IL23R	229	TH-17 like cell	CD4^+^ T cell
CD4_C09.CXCL13	272	Exhausted T cell	CD4^+^ T cell
CD4_C10.FOXP3	365	Peripheral Treg	CD4^+^ T cell
CD4_C11.IL10	176	Follicular regulatory T cells	CD4^+^ T cell
CD4_C12.CTLA4	1319	Tumor Treg	CD4^+^ T cell

### Pipeline for creation of the junction count matrix

We used an existing pipeline to create the junction count matrix (Fig. S1A). We first merged all the output of the SJ.out.tab files from the STAR aligner. Next, we retained junctions that were detected more than R_m_ reads in at least Cell_m_ cells (Cell_m_ = 10, R_m_ = 4, by default). Following this, we only retained the junctions that are only annotated to one gene. The reference gene annotation file is gencode.v27.primary_assembly.annotation.gtf. Lastly, we obtained the count matrix containing the junction read numbers in each cell.

### Description of software to detect DESJs

The software requires four inputs: junction count matrix (matrix A), junction annotation file (from the pipeline we developed), the uniquely mapped read number of each cell, and cell clustering information (Fig. 1A; Fig. S1A). First, we extracted junctions of a single gene (Gene1) from matrix A and normalized it with the number of uniquely mapped reads to obtain matrix C. Then, we performed iteration K-means clustering for cells in matrix C to identify outliers (standard deviation [SD (standard deviation)] < 0.2 and mean < 1 by default; precise steps are shown in Algorithm 1). Next, we normalized the remaining cells with all the junction read counts of Gene1 (matrix D). Finally, we used Limma-trend to detect the DESJs between groups. The software outputs a res.xls file including DESJs and junction expression heatmaps of each gene with DESJs. 
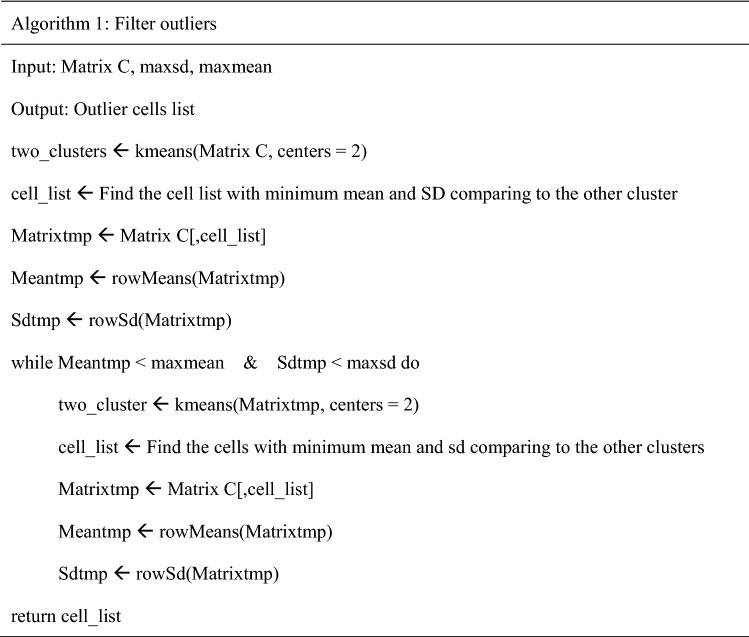


### Simulating scRNA-seq data and software evaluation

We simulated scRNA-seq data using a pipeline based on Spanki (v0.5.0)^32^. First, we chose 200 genes from human GTF files. Next, we selected two isoforms for each gene. Subsequently, we simulated reads per kilobase (RPK) value of 400 transcripts using a Perl script. The RPK value of a gene was constant, either at 25, 50, 100, or 200. However, the RPK ratio of two isoforms belonging to the same gene was reciprocal between two cells (cell from group A and group B respectively) for 100 genes. The cells from the same group were simulated with a similar RPK ratio of two transcripts belonging to the same gene. In addition, we set four levels of log2(RPK ratio) as 1, 2, 3, or 4 to represent the degree of isoform usage difference. In addition, we stimulated the dropout ratio as four levels: 0, 0.1, 0.2, and 0.4 by applying the simulator strategy of BRIE^16^. Finally, fastq files were generated using Spanki^32^ in error-free mode. We obtained 200 cells for each condition. A thorough description of the simulation can be found on github (https://github.com/lucky-Mendel/DSJ-detection-simulator). We then used these data to evaluate the performance of DESJ-detection with other software, including Outrigger, rMATS, DEXseq, and Limma-trend. The results provided by different tools at the level of isoforms, exons or events were aggregated to the gene level in order to compare the methods.

### Differentially expressed gene (DEG) analysis and gene set enrichment analysis

We used the Limma (v3.12) R package to analyze DEGs between two target clusters. Significant differences were identified by the following criteria: (1) false discovery rate (FDR)-adjusted *p*-value of *F* test < 0.01 and (2) the absolute value of log2(FC) > 2. Following this, we obtained the genes which were highly expressed in one group. We performed gene set enrichment analysis^33,34^ using a web-based tool provided by broad institute (http://www.gsea-msigdb.org/gsea/msigdb/annotate.jsp).

### Survival analysis

The Cancer Genome Atlas (TCGA) liver hepatocellular carcinoma (LIHC) data were used to assess the relationship between patient survival and individual genes, individual isoforms, and gene sets from specific cell clusters. We downloaded the gene expression and isoform count data from UCSC Xena^35^ (http://xena.ucsc.edu/) and retrieved clinical data from the Genomic Data Commons Data Portal (https://gdc-portal.nci.nih.gov/). Patients without immunotherapy treatment (*n* = 377) were included. First, the isoform read count data were normalized by isoform length and uniquely mapped read number of each patient. Then, to control for the influence of T cell level within each sample, the expression of selected genes and isoforms in the tumor were divided by the geometric mean expression of *CD3* genes. *CD3* gene expression was assigned as the arithmetic mean of the corresponding isoforms (*CD3D*, *CD3E,* and *CD3G*). Next, for each selected gene and isoform, we set the relative expression lower and upper threshold as the median ± 10% and the median absolute deviation (MAD), respectively. Samples with relative expression beyond these thresholds were retained and patients were divided into high and low expression groups. Statistical analyses were performed using the R package ‘‘survival’’.

### Trajectory inference

We used Monocle (version 2)^36^ to order CD8/CD4 T cells in pseudotime. The TPM value was converted into normalized mRNA counts by the “relative2abs” function in Monocle, and created an object with the parameter “expressionFamily = negbinomial.size”. Finally, the CD8^+^/CD4^+^ T cell differentiation trajectory was determined with the default parameters of Monocle.

### Definition of naïveness scores

Similar to Guo et al. (2018)^2^, we first identified the most significant genes between the naive T cluster (C06_CD4.CCR7) and other T clusters using a moderated *t*-test in the R package Limma (log2(FC) >  = 4 and FDR < 0.01). Then, we defined the naiveness score for CD8^+^ T cells as the average expression of these markers after z-score transformation (original value is log2(TPM + 1)). Finally, we calculated the significant level of the naiveness scores of cells from different clusters by *t* test.

### Clustering based on gene expression

To evaluate the difference between clustering based on gene expression and splicing, we applied Seurat (V3)^37^ to cluster cells in C06_CD4.CCR7 using the TPM expression matrix. The top 2000 variable genes were selected for downstream analysis. The Seurat parameters for PCs and resolution were set at 10–30 and 0.5–1, respectively. Finally, we utilized the adjusted rand index (ARI) to evaluate the similarity between clustering results of gene expression and ARHGAP15.

### Consent for publication

All the authors agreed to publish the work.

## Supplementary Information

Supplementary Figure 1.

Supplementary Table 1.

Supplementary Table 2.

## Data Availability

RNA-seq data of human T cells in fastq format was downloaded from EGD database with accession study title EGAS00001002072 and EGAS00001002791. The corresponding gene expression matrix was downloaded from the GEO database GSE108989 and GSE98638. Analysis code of such HCC data can also be found at https://github.com/liushang17/DESJ-detection.
