# Supplementary material for: Single-cell differential splicing analysis reveals high heterogeneity of liver tumor-infiltrating T cells
Source: Sci Rep. 2021 Mar 5;11:5325. doi: 10.1038/s41598-021-84693-w (PMC7935992; doi:10.1038/s41598-021-84693-w)
Supplement: Supplementary file 3 — Supplementary Table 2. [file 41598_2021_84693_MOESM3_ESM.pdf]

Single-cell differential splicing analysis reveals high heterogeneity of liver tumor-infiltrating T cells

Shang Liu[1,2,3], Biaofeng Zhou[1,2,3], Liang Wu[1,2,3], Yan Sun[1,2,3], Jie Chen[1,2], Shiping Liu[3,4*]

* Correspondence: liushiping@genomics.cn (Shiping Liu)

Affiliations:

[1]BGI Education Center, University of Chinese Academy of Sciences (UCAS), Shenzhen 518083, China.

[2]BGI-Shenzhen, Beishan Industrial Zone, Shenzhen 518083, China.

[3]Shenzhen Key Laboratory of Single-Cell Omics, China National GeneBank, Shenzhen 518120, China.

Emails:

Shiping Liu: liushiping@genomics.cn; Shang Liu: liushang@genomics.cn; Biaofeng Zhou: zhoubiaofeng@genomics.cn; Yan Sun: sunyan4@genomics.cn; Liang Wu: wuliang@genomics.cn; Jie Chen: chenjie4@genomics.cn

Supplementary figures

Figure S1

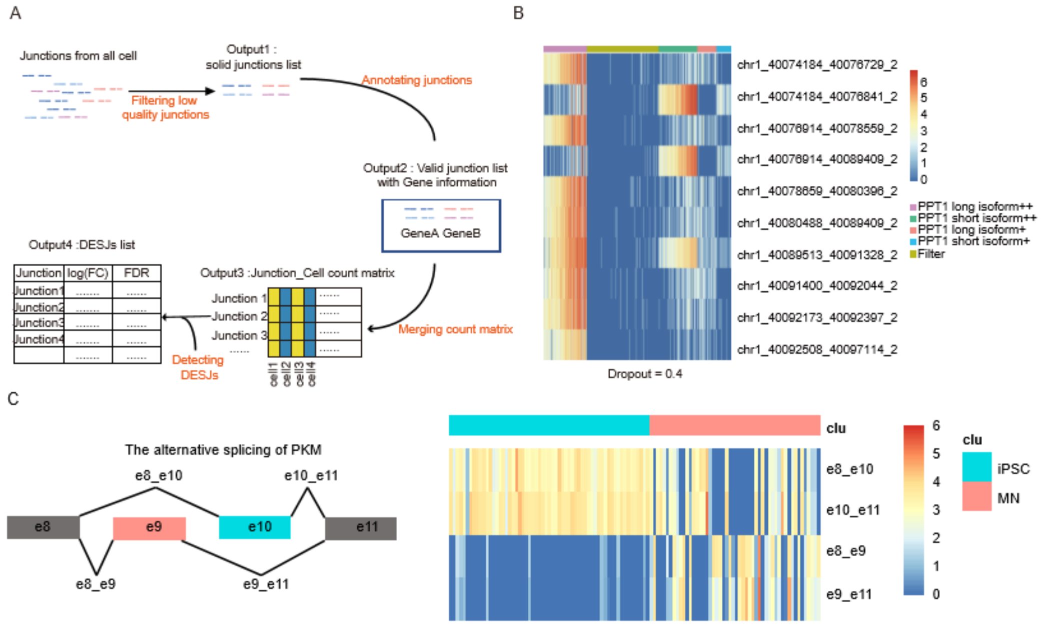

Figure S1. | A schematic of the pipeline for the creation of the junction count matrix and the performance of DESJ-detection on simulated data. A, a schematic of the preparation steps (see method for detailed information). **B**, Heatmap of five clusters identified by iterative K-means with all junctions of PPT1 from simulated data. The violet bar represents cells with high expression of the PPT1 long isoform. The green bar represents cells with high expression of the PPT1 short isoform. The pink bar represents a cell with moderate expression of the PPT1 long isoform, and the blue bar represents a cell with moderate expression of the PPT1 short isoform. The yellowish-brown bar represents a cell failing to reflect the composition of PPT1 isoforms. **C,** Differential inclusion of a mutually exclusive exon (MXE) alternative splicing (AS) event in PKM is observed in iPSC and MN. Left: the schematic of the MXE composed by exon 10 (e10) and exon 9 (e9). Right: the expression distribution of junction between cells from iPSCs and MNs.

Figure S2

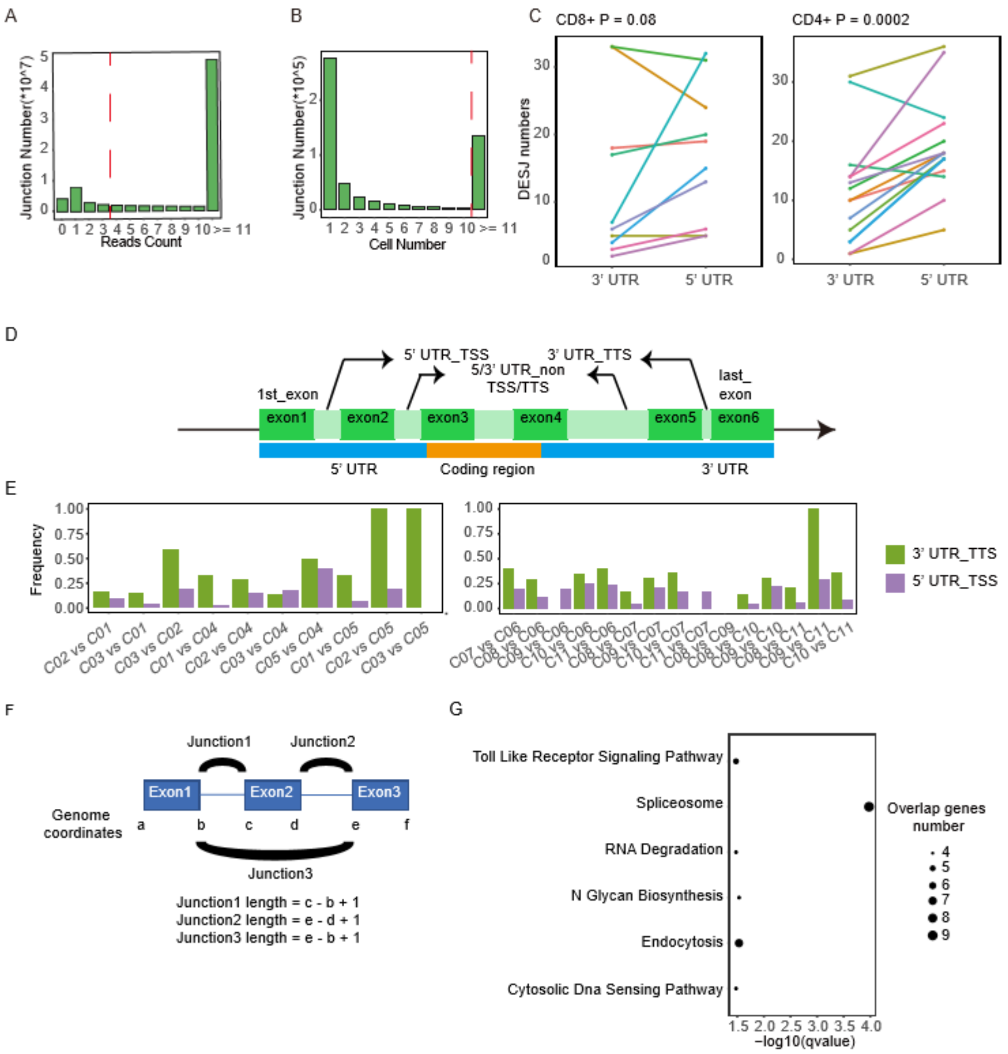

Figure S2. | Broad usage of DESJ in the UTR regions across T cell clusters. A, Read number distribution of all junctions from 5,063 cells. **B,** Cell number distribution of junctions with at least four supporting reads. **C,** Number of DESJ in the 3′ UTRs and 5′ UTRs across CD8+ and CD4+ T cells. These points represent T cell clusters. **D,** Structure of pre-mRNAs. 5′ UTR_TSS refers to junctions that may be associated with the TSS in 5′ UTRs. 5/3′ UTR_nonTSS/TTS refers to junctions that are not in the TSS and TTS. 3′ UTR_TTS refers to junctions that may be associated with the TTS in 3′ UTRs. **E,** The frequency of DESJ involved in the TSS and TTS in different UTRs across CD8+ and CD4+ T cells. **F,**. A schematic of the definition of junction length. **G,**.Results of KEGG pathway analysis of genes with differential splicing in the coding regions.

Figure S3

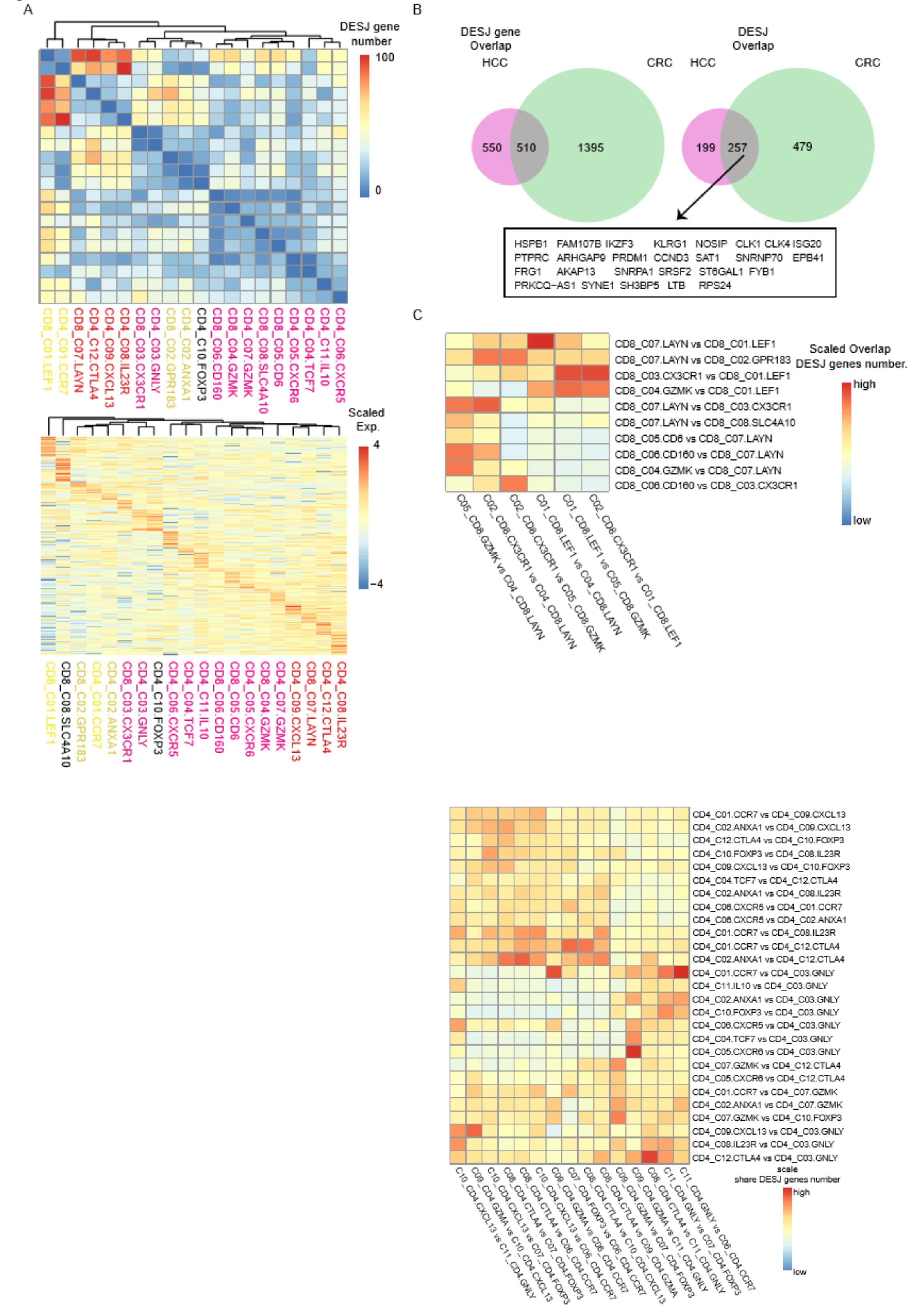

Figure S3. | T cell heterogeneity at the splicing level in CRC patients. A, Similar to Fig3 A, Upper: Heatmap of DESJ gene number between pairwise clusters across T cells. Below: z-score normalized mean expression of all DESJ in each T cell cluster. Clusters with different colors

highlight the T subtypes with different function. **B,** Venn plots show the overlap of DESJs and DESJ genes between CRC and HCC. The p value both are 0 according to chi-square test. **C,** Upper: the number of overlap DESJs between CRC and HCC. The DESJs are identified by each two CD8 T cell clusters. Below: the same as “Upper” in CD4 T cells clusters.

Figure S4

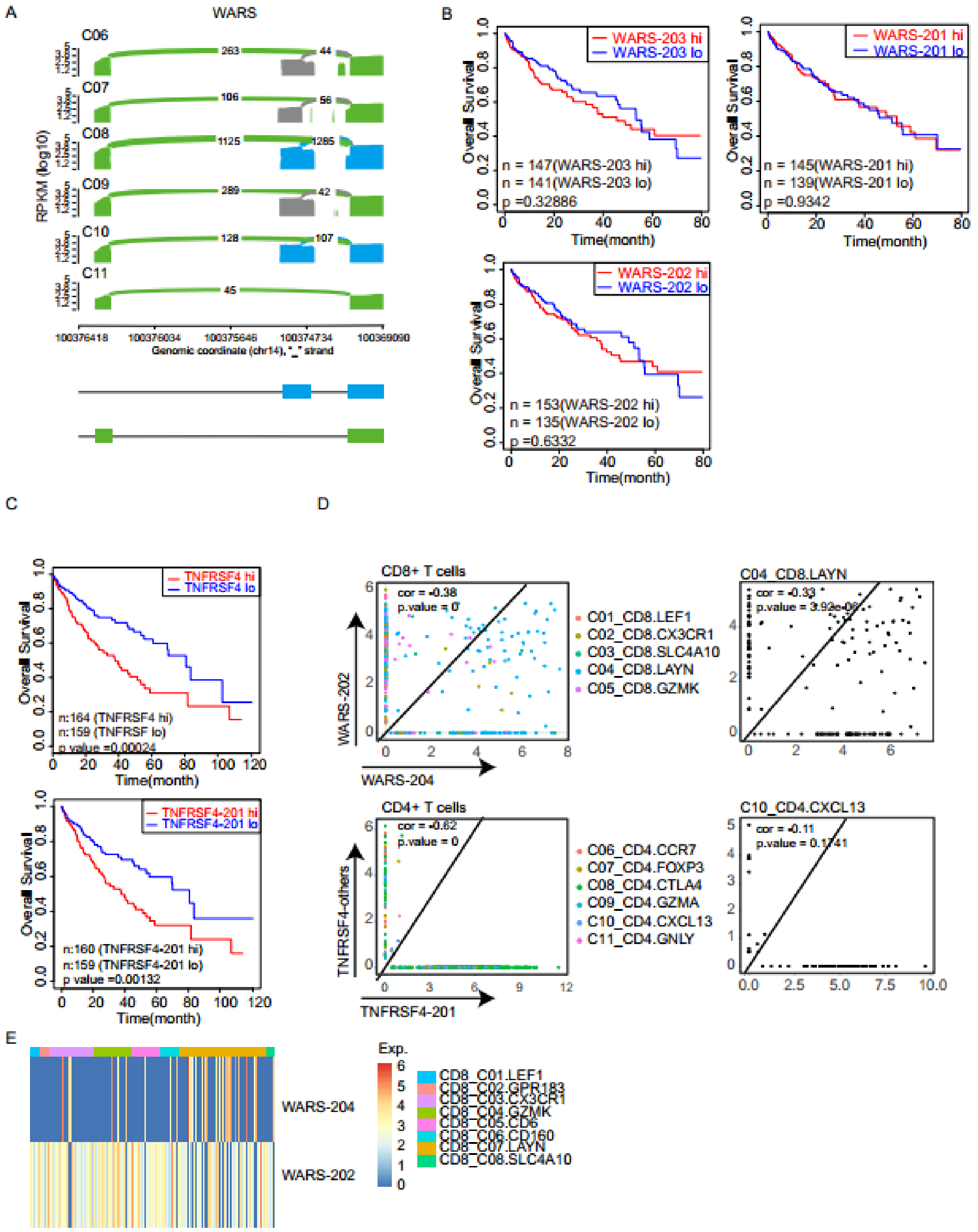

Figure S4. | T cell heterogeneity at the splicing level. A, Sashimi plot illustrating the read distribution of WARS in CD4+ T cells from patient P0508. WARS-204 is highly expressed in exhausted T clusters (C10_CD4.CXCL13) and Tumor Tregs (C08_CD4.CTLA4). **B,** DFS curve based on the TCGA HCC cohort showing the prognosis of WARS-201, WARS-202, and WARS-203. **C, A** DFS curve based on the TCGA HCC cohort showing the prognosis of *TNFRSF4* and TNFRSF4-201. **D,** A point plot shows the expression of WARS-202 and WARS-204 (upper) and TNFRSF4 (lower) across CD8+ T cells (upper) or CD4+ T cells (lower). **E,** Heatmap of normalized

expression of WARS-204 and WARS-201 across different T cells clusters in CRC patients.

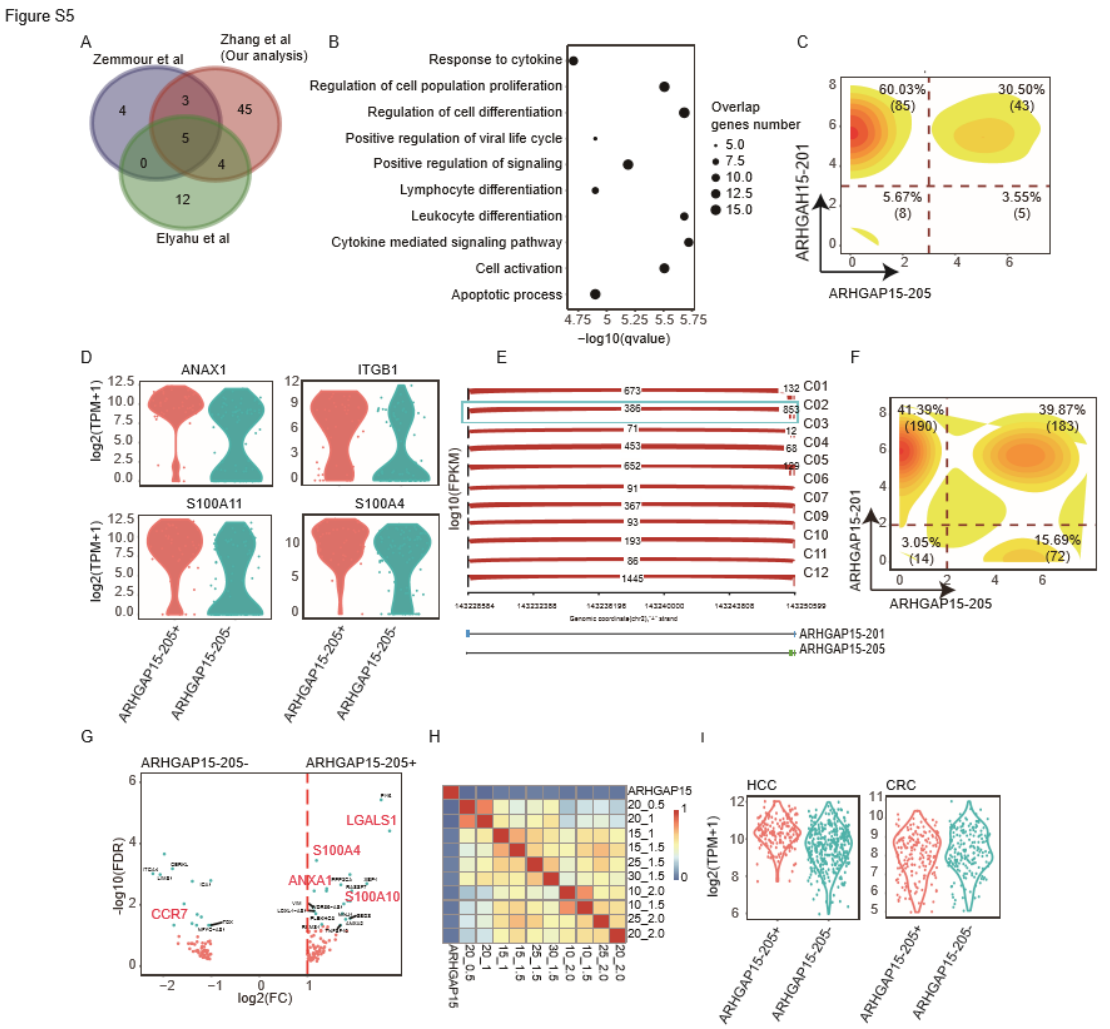

Figure S5. | A novel functional subpopulation in activation state identified by ARHGAP15-205. A, A Venn graph showing the overlap of activation associated genes identified in this study with those from a previous study by De Simone *et al.* (2016) (p = 2e-85) as determined by a hypergeometric test. **B,** Results of GO enrichment analysis of genes highly expressed in the ARHGAP15-205+ population. **C,** similar to Figure 4B, the bimodal distribution of ARHGAP15-205 shows the intrinsic heterogeneity in naïve T cells (C01_CD8.LEF1). **D,** Violin plots show the expression difference of activation markers including *S100A4, S100A11, ITGB1 and ANAX1* in the ARHAGP15-205^{-} and ARHGAP15-205^{+} C01_CD8.LEF1 subpopulations. E-G, Similar to Fig4A-C, the splicing pattern of ARHGAP15 in CD4 T cells clusters in CRC patients. H, A heatmap shows the ARI (adjust rand index) across clustering results from Seurat with gene expression and ARHGAP15 alternative splicing in C06_CD4.CCR7. The clustering from Seurat chose variable PCs among 10 to 30, and chose different resolutions from 0.5 to 1. **I,** The expression distribution of ARHGAP15 among the clusters from HCC and CRC.
